# Legislation for patient information leaflets in Iran: Focus on lay-friendliness

**DOI:** 10.15171/hpp.2018.37

**Published:** 2018-10-27

**Authors:** Razieh Azari, Marziyeh Khalilizadeh Ganjalikhani, Anahita Amirshoja’i

**Affiliations:** ^1^Faculty of Translation and Interpreting, University of Geneva, Geneva, Switzerland; ^2^Department of Foreign Languages, Higher Education Complex of Bam, Bam, Kerman, Iran

**Keywords:** Comprehension, Institutional context, Iran, Language, Lay-friendliness, Linguistics, Patient information leaflet

## Abstract

**Background:** Patient information leaflet (PIL) which accompanies medicinal products and informs patients about dosage, side effects, etc., is known as a tool which empowers patients to be more involved in making decisions related to their medications and health. In recent years, policy makers have paid much attention to PIL and its lay-friendliness i.e. being clear,explicit, and easy to read and understand. In various countries, PIL is known as a legal genre and must be prepared in understandable language. The present study aimed to situate PIL within its institutional context and investigate the importance is given to the lay-friendliness of PIL in Iran.

**Methods:** In this observational study, official website of Food and Drug Administration of the Islamic Republic of Iran (IFDA) was consulted. Iran’s rules and regulations, legal requirements,linked to PIL production and translation were introduced and discussed in relation to lay-friendliness..

**Results:** Limited legislation relevant for the production and translation of lay-friendly PIL was found. The IFDA has not issued any documents or guidelines which exclusively concern lay- friendliness of PIL. Any methods which can assess lay-friendliness in original and translated PILs were not observed.

**Conclusion:** The IFDA, the authoritative body in charge of legislation concerning PIL, has given limited importance to the language used in PIL and its lay-friendliness, witnessed by the limited legislation which is relevant for the production and translation of easy-to-understand PIL. It is not clear that how the IFDA assesses quality and lay-friendliness of original and translated PILs.

## Introduction

Patient information leaflets (PILs) which accompany medicinal products and inform patients about dosage, side effects, etc, are known as the bedrock of methods used to inform people about their medications and a tool which empowers patients and people to be more involved in making decisions related to their medications and health.^1,2^ In recent years, policy makers in various countries have paid much attention to PILs, especially their lay-friendliness i.e. being clear, explicit, and easy to read and understand. In various countries, PILs are known as a legal genre. In the European Union (EU), for example, PILs became a legal requirement in 1992 with Council Directive 92/27/EEC.^3^ Askehave and Zethsen^4^ stated that EU PILs are so-called mandatory genres, i.e. heavily, legally regulated genres. In Australia, PILs have been known as legally regulated genres since 1993.^5^ Moreover, PILs have to communicate complex health-related information to lay people, potentially the entire population of a country, thus being easy-to-understand, lay-friendly, is extremely important.^6^ For example, the EU has emphasized that PILs “must be written and designed to be clear, understandable and enable the users to act appropriately.”^7^

In Iran, PILs are governed by legislation as well.^8^ Despite the importance of PILs and the influence of legal requirements on lay-friendliness of PILs,^9,10^ to our knowledge, there has not been any research on Iran’s rules and regulations related to lay-friendliness of PILs. The present study sought to investigate the importance is given to the lay-friendliness of PILs in Iran.

PIL is a small leaflet which accompanies medicinal products and informs patients about what the drug is used for, dosage, possible side effects, etc. PILs are also sometimes referred to as patient package inserts, medical package leaflets, package leaflets, medicines information leaflets, consumer medicine information, written drugs information and written medicines information. In this study, for the sake of consistency, we have used the term Patient Information Leaflet; as Nisbeth Jensen^10^ mentioned, “this term refers to the function of the leaflet, and not merely its location.” Moreover, in this study, PIL refers to leaflets found inside medication packages providing information on dosage, possible side effects, etc and not leaflets for example found in doctors’ offices, hospitals, pharmacies, etc providing information on various diseases, procedures, and conditions.

Studies have shown that most patients read the PIL,^11^ and the “PIL is the most important source of information about a drug that patients have access to.”^12^ Raynor et al^13^ stated that the PIL is often the only source available when the patient actually takes the medicine. It is very important that patients are able to understand PILs; Nisbeth Jensen^6^ stated that PILs have to communicate complex health-related information to lay people, potentially the entire population of a country, thus lay-friendliness is extremely important. It is essential for informed decision-making and for the correct use of medication. If the receivers do not understand the communication, significant consequences will happen. In the following, the concept lay-friendliness was discussed.

As it was mentioned above, it is very important that patients are able to understand PILs. This requirement has been emphasized by policy makers and legislation in different countries. For example, in Australia, New Zealand, the United States, and the EU, PILs must be easy to understand for lay people and enable the users to act appropriately.^2^ The requirement was termed lay-friendliness in this study.

Scholars have used different terms for lay-friendliness. Gopferich^14^ used “comprehensibility”. Some used “readability”,^15-17^ “text complexity”,^18^ “text difficulty”,^19,20^ or “user-friendliness”.^4,21-23^ For the present study, we adopted “lay-friendliness” proposed by Nisbeth Jensen.^6,10^ There are several reasons for using the term *lay-friendliness* instead of other terms. For example, *user-friendliness* can be seen as vague because the notion of users is vague.^10^ *Readability* is associated with readability formulas and quantitative approaches and only offers a simplified view of the intricacies of text comprehension.^10^ Moreover, it suggests that it is an inherent text quality that can be assessed by looking at the document without taking into consideration who the receiver is.^10^ The term *lay-friendliness* “clearly shows that the text must be friendly or easy to understand for lay people, i.e. non-experts who do not have specialized knowledge.”^10^ The term *comprehensibility* and *complexity* can be seen as vague because they do not take into consideration who the receiver is.

## Materials and Methods

In this observational study, in order to find and investigate Iran’s rules and regulations, legal requirements, related to lay-friendliness of PILs, official website of Food and Drug Administration of the Islamic Republic of Iran (IFDA) was consulted. The IFDA is the authoritative body in charge of the legislation concerning PILs. All legislation concerning PILs is freely available on the IFDA website.

All legislation concerning PILs was identified and studied from beginning to end. In the next step, legislation related to lay-friendliness of PILs was identified. In the next sections the legislation was provided and discussed in detail.

It should be mentioned that this study exclusively looked at lay-friendliness in PILs from a linguistic perspective meaning that layout was not included.

## Results

In Iran, a pharmaceutical company must among other things provide a PIL in order to be granted marketing authorization of a pharmaceutical product.^24,25^ Thus, the production of PILs is a mandatory part of the granting of marketing authorization. Division of Pharmaceutical and Narcotic Affair (DPNA), part of the IFDA, is involved in assessing original and translated PILs.

According to *Regulations on Patient Information Leaflet*,^8^ PILs must be presented in Persian. This document also states what must be included in PIL and in which order. It means that both the content and the order of the content are legally regulated. Some of the main information which must be included in PIL is:

- Name of the product
- Pregnancy and breast-feeding
- Special warnings
- Appropriate precautions for use
- Forms of interaction with other medicinal products
- Necessary and usual instructions for proper use
- Dosage
- How often the medication should be taken
- Lists of contra-indications
- Storage condition
- Side effects

In the following, the *Regulations on Patient Information Leaflet* and other documents related to PIL were discussed in details.^8^

To produce PILs, a pharmaceutical company must consult *Regulations on Patient Information Leaflets*. This document is issued by the IFDA in 2010. The purpose of the document is promotion of patients and healthcare workers’ medicinal information as well as creation of favorable conditions for rational use of medicines.^8^ The document includes information to PIL producers which is presented in Table 1.

**Table 1:** Information to PIL producers provided by the *Regulations on Patient Information Leaflets*

**Information**
1.Writing principles of PIL
1.1.Language
1.2.Principles of writing
1.3.Font size and printing
2.Content of PIL
2.1.References
2.2.Consistency with Summary of Product Characteristic (SPC)
2.3.Headings
2.3.1.Name of the product
2.3.2.Identification of the medicine
2.3.3.Medicine indication
2.3.4.General instructions for patients
2.3.5.Lists of contra-indications
2.3.6.Pregnancy and breast-feeding
2.3.7.Warnings
2.3.8.Precautions
2.3.9.Interaction with other medicinal products
2.3.10.Dosage and usual instructions for proper use
2.3.11.Side effects
2.3.12.Storage condition
2.3.13.Signs of damaged medicines
2.3.14.Last date on which the leaflet was prepared and revised
2.3.15.Name, address, telephone number, fax number, E-mail address, and website of the pharmaceutical company or authorized agent in Iran

This document also includes regulations related to lay-friendliness. Following Regulation 1 (2),^8^ PIL must be prepared in simple, clear, and understandable language. The use of complex scientific terminology, long sentences, and acronyms without enough explanation must be avoided.^8^ Also, punctuation must be used and must be clear.^8^

Following Regulation 2 (2),^8^ PIL must be prepared in simple, clear, and understandable language and must be in accordance with the Summary of Product Characteristics (SPC).

Following Regulation 2 (3) (5),^8^ lists of contra-indications must be stated in understandable language. Also, it is recommended to use specific sentences.^8^

In Regulation 2 (3) (7),^8^ it is recommended to use imperative sentences for stating contents related to Warnings.

Following Regulation 2 (3) (10),^8^ dosage and usual instructions for proper use must be stated in clear language. Full details must be stated. Also, it is recommended to use specific sentences.^8^

In Regulation 2 (3) (4, 9, 11, 12, 13),^8^ it is recommended to use specific sentences.

To produce leaflets for biological products, a pharmaceutical company must consult *Packaging of Biologic Products*. This document is issued by the IFDA in 2009. The document includes rules on “outer package”, “container”, and “biological products brochure”. In this section, only the rules related to “biological products brochure” are concerned. Here brochure is identical to the PIL. Based on the document, “every biological product must be provided with a brochure or manual.”^26^ Also, “the Persian version of the brochure must be provided in the first cargo of products.”^26^ It is mentioned that all pages of the brochure must be provided with the confirmation of technical connoisseur and a related member of faculty. In this document, use of specific sentences is recommended, but any rules and regulations related to lay-friendliness of brochures were not observed.

In Iran,^24^ in order to be granted marketing authorization of an imported pharmaceutical product, an authorized agent, any legal entity that holds an exclusive agency of the product license holder (PLH) or marketing authorization holder (MAH), must among other things provide Persian translation of PIL. The authorized agent in Iran will be held responsible for the contents of the Persian PIL as for its conformity with the manufacturer’s leaflet.^24^ The authorized agent must submit a sample of the Persian translation of the PIL and packaging as stated in *Appendix 8: Regulations on Packaging of Imported Pharmaceutical Products*.^24^ As previously mentioned, the DPNA is involved in assessing original and translated PILs.

As it was mentioned above, the authorized agent must consult *Appendix 8: Regulations on Packaging of Imported Pharmaceutical Products* to produce Persian translation of a PIL.^24^ This document includes regulations on labelling, packaging, and PILs of imported pharmaceutical products. In this section, only the regulations related to PILs are concerned. Based on the document, “printings on and in the package should be both in English and Persian.”^24^ The PIL shall be presented in both Persian and English.^24^ The second language of PILs,^24^ other than Persian should be English; other languages (German, French, etc) are not acceptable. According to the document, “the importing company is bound to supply the first consignment of pharmaceutical products along with appropriate Persian translation [of PIL].”^24^ It is also stated in the *Packaging of Biologic Products*.^26^ PIL must be fully translated.^24^ In case the pharmaceutical product includes 2 leaflets for the patient and the physician,^24^ the Persian translation of the PIL is sufficient, but the physician leaflet in English shall be presented along with the product as well. Name, address, telephone number, fax number, and E-mail address of the authorized agent in Iran must be included in the PIL.^24^ In this document, any rules and regulations related to lay-friendliness of translated PILs were not observed.

## Discussion

The present study served the purpose of situating PIL within its institutional context and investigating the importance is given to lay-friendliness of PIL in Iran. To achieve the objectives of the study, legal requirements linked to PIL production and translation in Iran were introduced and discussed in relation to the lay-friendliness.

As it was mentioned, PILs in Iran are governed by legislation and must be prepared in simple, clear, and understandable language here termed lay-friendly.^8^

When investigating the *Regulations on Patient Information Leaflets*,^8^ the main document a pharmaceutical company must consult for producing PILs, it becomes evident that limited importance is given to the lay-friendliness of PILs. Only four regulations related to lay-friendliness of PILs were observed. Moreover, no references to researches or studies are provided to show that the IFDA checked the document and its regulations to see whether they can optimize lay-friendliness of PILs. It means that the document and its regulations have not been supported by any evidence. More studies are needed to investigate whether this document can help a pharmaceutical company to produce a simple, clear, and understandable PIL.

When investigating the *Packaging of Biologic Products*,^26^ it becomes evident that no importance is given to the lay-friendliness of PILs. In this document, use of specific sentences is recommended, but any rules and regulations related to lay-friendliness of PILs were not observed. Like previous document, no references to researches or studies are provided to show that the IFDA checked the document and its regulations to see whether they can optimize lay-friendliness of PILs. More studies are needed to investigate whether this document can help a pharmaceutical company to produce a simple, clear, and understandable PIL.

Regarding translation of PIL, only one document, *Appendix 8: Regulations on Packaging of Imported Pharmaceutical Products*,^24^ was found. In this document, any rules and regulations related to lay-friendliness of translated PILs were not observed. Again, no references to researches or studies are provided to show that the IFDA checked the document and its regulations to see whether they can optimize lay-friendliness of translated PILs. More studies are needed to investigate whether this document can help pharmaceutical companies or an authorized agent to produce a simple, clear, and understandable PIL.

Moreover, the IFDA has not issued any documents or guidelines which exclusively concern lay-friendliness of PIL. Any methods which can assess lay-friendliness in original and translated PILs were not observed. There are various methods for assessing lay-friendliness in PILs including user-testing. User-testing is one of the main methods of assessment. For example, in the EU, mandatory user-testing of PILs was implemented for all marketing authorizations granted after October 30, 2005.^27^ According to Article 59 (3), “the package leaflet shall reflect the results of consultations with target patient groups to ensure that it is legible, clear and easy to use.”^27^ The IFAD has not considered any assessment methods.

As previously mentioned, the DPNA is involved in assessing original and translated PILs. It is not clear that:

1. How they check the quality of original and translated PILs;
2. How they assess whether a PIL prepared in simple, clear, and understandable language i.e. lay-friendly;
3. What kind of scale they use to assess original and translated PILs;
4. What kind of scale they use to assess lay-friendliness of original and translated PILs;
5. What kind of scale they use to assess whether a PIL is fully translated.

The legal requirements related to lay-friendliness of PILs could be improved in several ways. Health policy makers in general and the IFDA in particular could evaluate the documents to see whether they can optimize lay-friendliness of PILs. Health professionals, health education and promotion specialists, and those who are interested in the field could collaborate and carry out some researches and studies to response the question. The IFDA could collaborate with Translation Studies scholars to improve the legal requirements related to lay-friendliness of translated PILs. The IFDA could consider (1) legal requirements which exclusively concern lay-friendliness of PIL and (2) an assessment method such as user-testing which assesses lay-friendliness in original and translated PILs. International experiences such as Australia, New Zealand, and the EU could be considered. Related scholars could investigate and propose assessment methods. The IFDA could consider (1) teams consist of health professionals, health sociologists, linguists, and professional translators and (2) clear and defined procedures, timeline, assessment scales for checking and assessing lay-friendliness of original and translated PILs. The results of applied legal requirements related to lay-friendliness of PILs could be investigated by related scholars which leads to improvements of lay-friendliness in PILs.

## Conclusion

Despite the importance of PIL and its lay-friendliness, IFDA, the authoritative body in charge of legislation concerning PIL, has given limited importance to the language used in PIL and its lay-friendliness, witnessed by the limited legislation which is relevant for the production and translation of easy-to-understand PIL. Even though Iran’s legislation states that PIL must be prepared in understandable language, any methods which can assess lay-friendliness in original and translated PIL were not observed. It is not clear that how the IFDA assesses quality and lay-friendliness of original and translated PILs. More studies are needed to investigate whether the legal requirements can help a pharmaceutical company to produce a simple, clear, and understandable PIL.

## Ethical approval

Not applicable

## Competing interests

The authors declare that they have no competing interests.

## Funding

The authors did not receive any funding for this study.

## Authors’ contributions

RA developed the study concept and design, contributed to data collection, analysis, and interpretation, and drafted the manuscript. MKG contributed to data collection and analysis. AA contributed to evaluation, edition, and revision of the manuscript. All authors reviewed and provided intellectual feedback on the manuscript. All authors read and approved the final manuscript.
